# Neuromuscular Control Modelling of Human Perturbed Posture Through Piecewise Affine Autoregressive With Exogenous Input Models

**DOI:** 10.3389/fbioe.2021.804904

**Published:** 2022-01-21

**Authors:** Andrea Tigrini, Federica Verdini, Marco Maiolatesi, Andrea Monteriù, Francesco Ferracuti, Sandro Fioretti, Sauro Longhi, Alessandro Mengarelli

**Affiliations:** Department of Information Engineering, Università Politecnica Delle Marche, Ancona, Italy

**Keywords:** perturbed posture, system identification, piecewise affine autoregressive with exogenous inputs, neuromuscular control, cognitive load

## Abstract

In this study, the neuromuscular control modeling of the perturbed human upright stance is assessed through piecewise affine autoregressive with exogenous input (PWARX) models. Ten healthy subjects underwent an experimental protocol where visual deprivation and cognitive load are applied to evaluate whether PWARX can be used for modeling the role of the central nervous system (CNS) in balance maintenance in different conditions. Balance maintenance is modeled as a single-link inverted pendulum; and kinematic, dynamic, and electromyography (EMG) data are used to fit the PWARX models of the CNS activity. Models are trained on 70% and tested on the 30% of unseen data belonging to the remaining dataset. The models are able to capture which factors the CNS is subjected to, showing a fitting accuracy higher than 90% for each experimental condition. The models present a switch between two different control dynamics, coherent with the physiological response to a sudden balance perturbation and mirrored by the data-driven lag selection for data time series. The outcomes of this study indicate that hybrid postural control policies, yet investigated for unperturbed stance, could be an appropriate motor control paradigm when balance maintenance undergoes external disruption.

## 1 Introduction

Although upright posture maintenance could seem a straightforward task, its study can reveal complex control mechanisms aimed at preventing falls (Horak and Nashner, 1986; Suzuki et al., 2012). Posture has been studied using different experimental protocols and different data sources that can be grouped in two main areas: unperturbed and perturbed posture (Horak and Nashner, 1986; Peterka, 2002; Visser et al., 2008). The former task refers to the quiet upright stance in which the only external force considered is the gravitational one (Peterka, 2000, 2002; Morasso et al., 2019), while different internal factors that can potentially promote instability may arise, i.e., postural noise or change in the visual field (Conforto et al., 2001; Berencsi et al., 2005). Due to the convenience in many aspects of its study, including simple and well-assessed measuring protocols, unperturbed posture has played a key role in developing neuromuscular models of the central nervous system (CNS). However, different applications can also be found in the clinical scenario to study pathologies potentially affecting the CNS (Corradini et al., 1997; Fioretti et al., 2010).

Conversely, perturbed posture refers to those tasks in which not only gravitational pull acts on the body, but other additive external disturbances, i.e., support base movements, external forces, and vibrations, are employed in perturbing the upright stance (Horak and Nashner, 1986; Patel et al., 2009; Engelhart et al., 2014). Such disturbances necessarily challenge the CNS, which responds with motor commands to counteract those potentially dangerous sources of instability (Horak and Nashner, 1986). Among all of the possible types of perturbation, support base movement has constituted one of the most employed due to its capability in reproducing everyday life experiences (Horak and Nashner, 1986; Nanhoe-Mahabier et al., 2012; Mengarelli, 2017). Additive factors were in general introduced to get closer to a real scenario; namely, subjects are asked to close their eyes (visual deprivation) or perform mental counting (cognitive load) while they undergo the experiment (Albertsen et al., 2017). The responses that the CNS actuates have been assessed through different signals such as the center of pressure (CoP), sway angles, and surface electromyography (sEMG). Moreover, as described in Jacobs and Horak (2007), evidences confirm that evoked postural responses to external perturbations may involve also higher control centers, e.g., cortical involvement.

As suggested by many works (Peterka, 2002, 2000; Suzuki et al., 2012; Nomura et al., 2013; Morasso et al., 2019; Zhang et al., 2014, 2016), a modeling perspective that accounts for biomechanical quantities (sway angles, CoP, and joint torques) can be considered a fundamental core in postural analysis, for explaining possible CNS control mechanisms. In this context, system identification provided a valuable way to model the CNS activity as effectively proven when the ARMAX was employed to analyze the postural control rearrangement in patients affected by multiple sclerosis and hemiparesis (Corradini et al., 1997). The aforementioned study considered an unperturbed posture protocol, while other identification procedures were studied for perturbed posture protocols (Engelhart et al., 2014; Ersal et al., 2014). In order to perform closed-loop identification of the CNS controller, in Engelhart et al. (2014), an experimental device was presented and used for neuromuscular control identification purposes, highlighting the importance of challenging balance maintenance with external forces. A relation between torques (controller output) and sway angles was estimated following de Vlugt et al. (2003), which essentially made use of the partial coherence to estimate the neuromuscular frequency response function (FRF).

The experimental protocol and the device employed in Engelhart et al. (2014) have been specifically designed and realized to provide a stance perturbation by applying an impulsive external force to the upper and lower limbs, in order to obtain a disturbance time series minimally correlated to joint torques, which represent the output of the balance regulatory activity. This got closer the hypothesis behind the identification procedures to the properties of the measured data. However, in the clinical scenario, support base movement still represents the most common choice for investigating upright stance maintenance after external perturbations (Dimitrova et al., 2004; Nardone et al., 2007; Visser et al., 2008), but from a modeling point of view, surface translations present some issues that need to be highlighted. For instance, the acceleration of the platform induces an inertial disturbance at the center of mass (COM) that cannot be independent with respect to the control torques. In addition, linear approximations, commonly employed in the modeling of the neural controller, may not hold anymore, eventually revealing a nonlinear behavior of the upright balance process (Tigrini et al., 2019).

A possible way to deal with such nonlinearity is the use of discontinuous control models. As highlighted by Morasso and co-workers (Morasso et al., 2019), much evidences suggest a discontinuous or hybrid nature of the postural feedback control, and this aspect was treated starting from a modeling perspective in a few fundamental works that dealt with the quiet stance (Asai et al., 2009; Suzuki et al., 2012). At the same time, literature manifested an increasing interest in hybrid system identification, and different methodologies have been developed (Lauer and Bloch, 2019). Therefore, investigating whether a hybrid control policy can be recognized as a plausible physiological control mechanism, also when the upright stance is tampered with external disruptions, may be a valuable choice in order to deepen the understanding of the CNS motor regulatory mechanisms. However, the idea of modeling neural controllers with hybrid properties has not yet been applied to analyze human balance maintenance in a perturbed scenario.

Hybrid systems are dynamical systems arising from the interaction between continuous and discrete dynamics and can be used to model physical phenomena characterized by a discontinuous behavior (Paoletti et al., 2007). Piecewise affine (PWA) models represent hybrid systems obtained by the partition of the state-input domain into non-overlapping polyhedral regions and then considering affine subsystems for each region. Such kinds of models were suitable for deriving hybrid models from the data and also for nonlinear system identification (Paoletti et al., 2007). In this work, the role played by the neuromuscular controller has been identified, through the use of piecewise affine autoregressive (AR) with exogenous input (PWARX) models (Nakada et al., 2005; Paoletti et al., 2007), where multiple sources of information can contribute to the model building. More in detail, the upright stance has been modeled as a single inverted pendulum (SIP) (Morasso et al., 2019), and the control torque, obtained using kinematic and dynamic data, has been modeled through the use of the sway angle and sEMG data as exogenous inputs. Data acquired from ten subjects who underwent sudden perturbations of balance, involving also visual deprivation and cognitive load, have been employed; and PWARX model identification was able to capture the different strategies adopted by the CNS in managing the information to generate regulatory commands (Albertsen et al., 2017).

The paper is organized in the following way: in Section 2, the details about the experimental data acquisition, the biomechanical modeling of the upright stance, and the system identification procedure are provided. The results are reported in Section 3 and discussed in Section 4, while final remarks in Section 5 end the paper.

## 2 Methods

### 2.1 Experimental Protocol

Ten healthy subjects were recruited for this study. None of them was affected by neurological or musculoskeletal disorders that may affect their abilities in balance maintenance. Subjects were informed regarding each phase of the experimental protocol, and they gave written informed consent prior to the beginning of the test. The study was undertaken in compliance with the ethical principles of the Declaration of Helsinki and was approved by the local ethics committee.

Each participant was instrumented with 26 reflective markers placed on anatomical landmarks following Leardini et al. (2007). Additional markers were placed on the platform corners, in order to track translational movements. All the experiments were acquired through a six-camera optical motion analysis system (BTS Elite, Milan, Italy).

A Kistler force platform was used to collect dynamic data, namely, the CoP and ground reaction forces. Further, muscle activity was collected through sEMG. The signals were recorded bilaterally from the tibialis anterior (TA) and the gastrocnemius medialis (GA). All data were synchronously acquired, and kinematics was acquired with a sampling frequency of 100 Hz, while sEMG and force plate data were sampled at 1,000 Hz.

After being instrumented, participants stood on a servo-controlled movable platform, waiting for the rise of the external disturbance. Each perturbation consisted of a backward horizontal displacement (5 cm) of the base of support, with a time duration of 0.3 s. Each subject underwent a two-stage experiment. In the first stage (training), the participants performed ten trials with eyes open to avoid the first trial effect and account for the habituation rate (Nanhoe-Mahabier et al., 2012). Then, in the second stage, three further trials were performed: the first one with eyes opened (EO), the second one with eyes closed (EC), and the third with each subject standing on the platform with eyes opened counting back from 100. The latter is referred to as dual task (DT) and was designed to investigate how the postural control reflex may change in the presence of a cognitive task. The time at which the platform motion starts remained unknown for the subject in both stages of the experiment. Each trial was accepted whether the subject maintained both feet on the platform for the entire duration of the record, without step responses. Otherwise, the trial was discharged and repeated.

### 2.2 Biomechanical Model

Modeling perturbed posture with support base shift requires some physical considerations, while in Engelhart et al. (2014), the perturbing device can apply direct forces to the human subject; in support base shift, this does not happen. The support base acceleration induces an inertial force coupled with the gravity pulling to the body. This ensemble of forces generates disturbance torques at the lower limb joints, eventually perturbing the human upright stance. It is possible to model the physics mentioned above in the sagittal plane employing a multi-link inverted pendulum on a moving platform (Tigrini et al., 2019).

**FIGURE 1:**
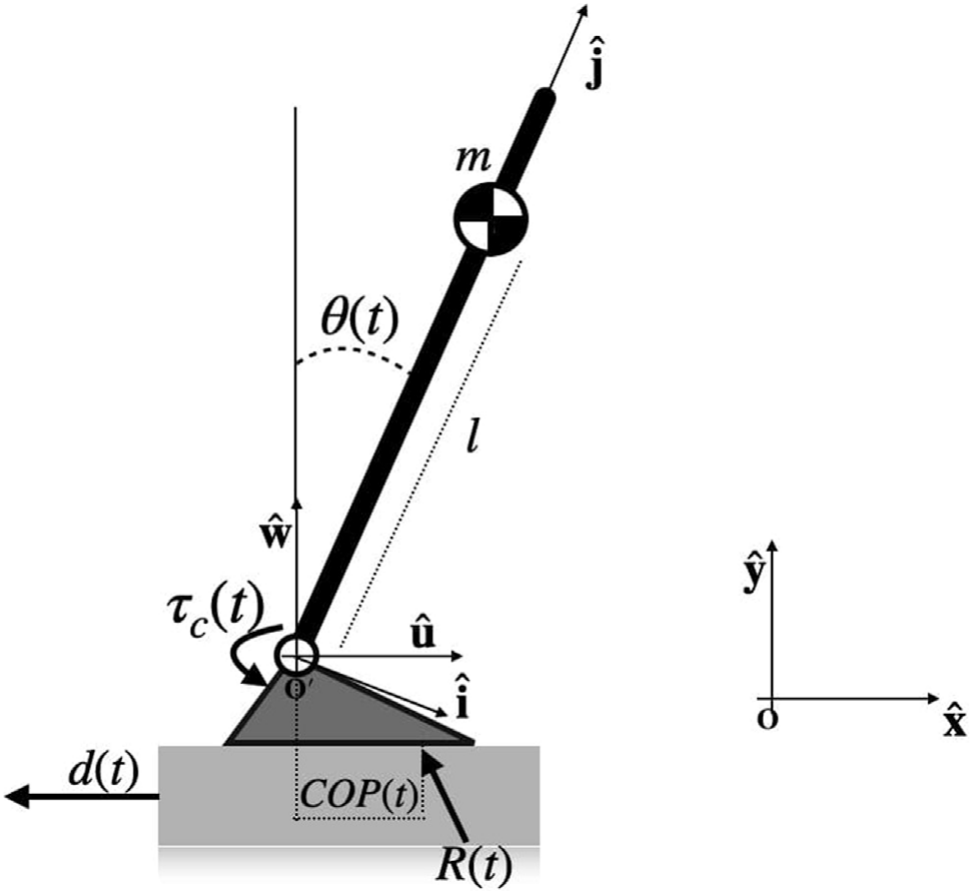
Biomechanical model of the upright stance. The generalized coordinates and the reference frames are also reported, together with the external forces acting on the system and the internal control torque, modeled as a lumped control action acting at the ankles.

However, as highlighted in the previous section, the magnitude of the disturbance employed and the subjects’ training phase permitted to assume that the balancing response was mainly based on the ankle strategy (Horak and Nashner, 1986), enforcing the validity of a single-link inverted pendulum model for describing the biomechanics of the motor task. Hence, in this study, a simple model, i.e., single-link inverted pendulum standing on a support base (Figure 1), was used to describe the mechanics of the upright stance, where the whole body is considered as a unique rigid rod hinged at the level of the ankle (Figure 1) and the feet are modeled with no inertia and assumed to be fixed with the platform.

In order to describe the human stance mechanics, let us assume the following three reference frames (RFs): $R_{g}=\left\{O,\;\hat{x},\;\hat{y}\right\}$ representing the global one; $R_{p}=\left\{O^{\prime },\;\hat{u},\;\hat{w}\right\}$ representing the platform RF whose origin coincides with the ankle joint (*O*′); and *R*_*b*_ denotes the pendulum RF with the origin in *O*′. The mechanical system has two degrees of freedom and can be described by **q**(*t*) = [*x*(*t*) *θ*(*t*)], where *x*(*t*) represents the backward displacement along the horizontal axis (Figure 1), and *θ*(*t*) is the sway angle of the rod with respect to the vertical axis $\hat{w}$ . The mass *m* of the pendulum can be considered to be concentrated in a single point. In order to derive the equation of the system, conservation of the angular momentum can be applied:

$$\frac{dL}{dt}=\overset{n}{\underset{k=1}{\sum }}M_{k_{/O}}$$ (1)

where the term $\frac{dL}{dt}$ is the rate of change of the angular momentum of the mechanical system and $\sum_{k=1}^{n}M_{k_{/O}}$ represents the sum of all moments generated by external forces with respect to the origin of *R*_*g*_. In this case *n* = 2, since the moments acting on the system are as follows: **M**_*g*_ caused by gravitational field acting on the COM *m* and the global torque applied at the ankle, generated by the ground reaction force **R** applied at the CoP (Figure 1). Then, it is possible to obtain the equation of the SIP:

$$M_{a}\left(t\right)=ml^{2}\ddot{\theta }\left(t\right)-mglsin\left(\theta \left(t\right)\right)+\dot{d}\left(t\right)mlsin\left(\theta \left(t\right)\right)\dot{\theta }\left(t\right)-\ddot{d}\left(t\right)mlcos\left(\theta \left(t\right)\right)$$ (2)

where the term *M*_*a*_(*t*) indicates the total torque generated at the ankle, which depends on the linear displacement *d*(*t*), angular displacement *θ*(*t*), and their time derivatives. The angle *θ*(*t*) will be treated as controlled variable and the linear acceleration $\ddot{d}\left(t\right)$ as disturbance for the system. The element that depends on the linear velocity $\dot{d}\left(t\right)$ and angular velocity $\dot{\theta }\left(t\right)$ is a Coriolis term.

The experiment presented in this study consistently differs from those posturography protocols with no movements of the support platform (Corradini et al., 1997; Engelhart et al., 2014). The term *M*_*a*_(*t*) contains two components, i.e., the neuromuscular (internal) control torque and the disturbance torque. The latter can be considered as the sum of the gravity pull and the inertial term induced by the disturbance $\ddot{d}\left(t\right)$ . Hence, to retrieve the control torque at the ankle, the following decomposition has been taken into account:

$$\left\{\begin{array}{cc} \tau_{d}\left(t\right) & =-mglsin\left(\theta \left(t\right)\right)+\dot{d}\left(t\right)mlsin\left(\theta \left(t\right)\right)\dot{\theta }\left(t\right)-\ddot{d}\left(t\right)mlcos\left(\theta \left(t\right)\right)\\ \tau_{c}\left(t\right) & =M_{a}\left(t\right)-\tau_{d}\left(t\right) \end{array}\right.$$ (3)

where *M*_*a*_(*t*) is the external ankle torque, obtained as the cross product between position vector of the *CoP*(*t*) with respect to the ankle joint and the ground reaction force **R**(*t*) (Figure 1). *τ*_*d*_(*t*) is the torque generated by the disturbances, while *τ*_*c*_(*t*) is the internal control torque modeled as the difference between the measured *M*_*a*_(*t*) and the disturbance torque *τ*_*d*_(*t*), derived by the model.

### 2.3 Data Cleaning

**FIGURE 2:**
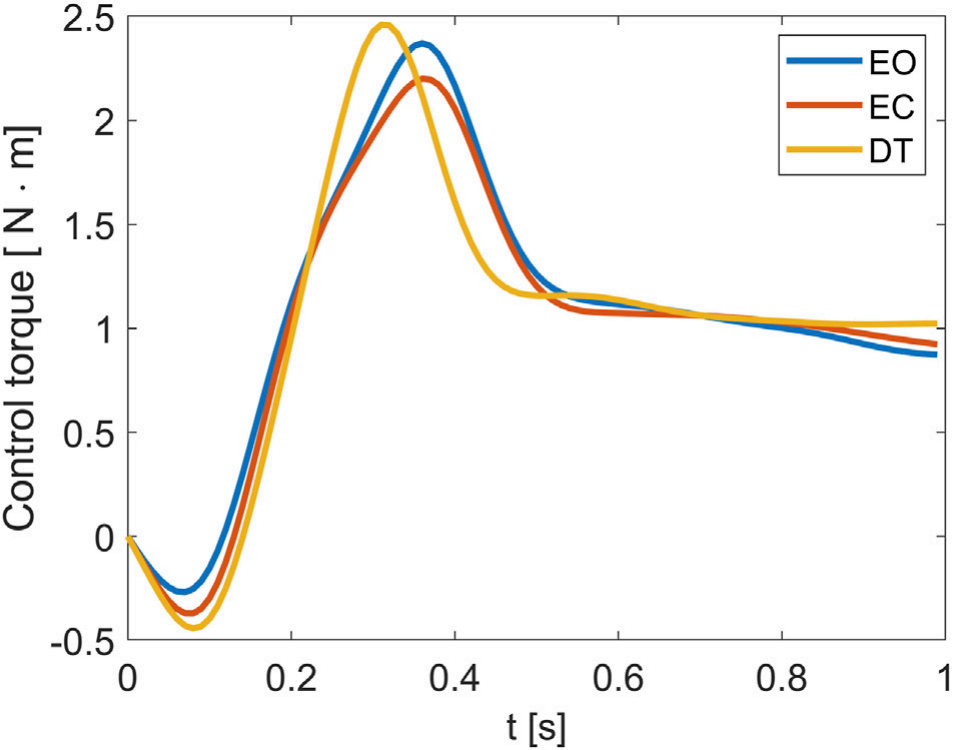
Control torque *τ*_*c*_(*t*) for a representative subject measured in the three experimental conditions.

For each subject, data series of 1 s are considered, starting from the beginning of the perturbation for the EO, EC, and DT conditions. This was motivated by the aim of examining the control dynamics taking place during the transient phase of the response, avoiding the possible voluntary control effects arising whether greater temporal epochs are taken into account. This choice appears in line with other previous studies dealing with the same topic (Diener et al., 1988; Visser et al., 2008; Allum et al., 2011; Nanhoe-Mahabier et al., 2012). Kinematic data are filtered with a second-order zero-phase low-pass Butterworth filter with a 10-Hz cutoff frequency. Kinetic data are low-pass filtered in the same way, with a cutoff frequency of 15 Hz, and then detrended and down-sampled at 100 Hz. By using inverse dynamics, following the decomposition presented in Eq. 3, it is possible to retrieve *τ*_*c*_(*t*) as the difference between *M*_*a*_(*t*) and the disturbance torque *τ*_*d*_(*t*), obtained by the model. A graphical representation of the control torque *τ*_*c*_(*t*) of a representative subject, for the three considered conditions, is reported in Figure 2.

Regarding the sEMG data, the GA and TA signals of the dominant leg are taken into account and band-pass filtered between 30 and 450 Hz. Then, the root mean square (RMS) is computed and down-sampled at 100 Hz. At the end of the data cleaning step, for each subject and for each condition, 4 time series made by 100 samples were used to model the neuromuscular control, namely, the control torque *τ*_*c*_(*t*), the sway angle *θ*(*t*), and the RMS of GA and TA.

### 2.4 Identification Procedure and Analysis

PWA models are a representation of hybrid systems, and according to Paoletti et al. (2007), they can be obtained through partitions of the regression space into a finite number of non-overlapping convex polyhedral regions, where linear/affine subsystems are identified. Therefore, such piecewise-defined models are often used to approximate a single nonlinear continuous behavior by a collection of linear or affine sub-models, each of which is valid only in a particular region. Let us consider a system in the input–output (I/O) form:

$$y_{k}=f\left(x_{k},e_{k}\right)$$ (4)

where $y_{k}\in R^{p}$ is the output vector, $x_{k}\in R^{n_{d}}$ is the regression vector, and $e_{k}\in R^{p}$ is the noise vector, which includes lagged values of the input ***u***_*k*−*i*_ and the output ***y***_*k*−*i*_. Following this representation, for a hybrid system, the following holds:

$$y_{k}=f_{\sigma \left(k\right)}\left(x_{k},e_{k}\right)$$ (5)

where ***y***_*k*_, ***x***_*k*_, and ***e***_*k*_ are as in Eq. 4, and the discrete state *σ*(*k*) ∈ {1, *…* , *s*} selects the active sub-model ${\left\{f_{j}\right\}}_{j=1}^{s}$ at time *k*, where *s* is the number of sub-models. The discrete state *σ*(*k*) depends on the continuous regression vector ***x***_*k*_, i.e., *σ*(*k*) = *j* if $x_{k}\in X_{j}$ , where $X_{j}$ are regions that form a partition of the whole regression space X . Hybrid system identification, in the form given by Eq. 5, requires a parameterization in order to identify an opportune I/O data relation. Thus, Eq. 5 becomes

$$y_{k}=f_{\sigma \left(k\right)}\left(x_{k}\left[\theta_{\sigma \left(k\right)}\right],e_{\sigma \left(k\right)},\theta_{\sigma \left(k\right)}\right)$$ (6)

which implies the determination of all the parameter vectors ${\left\{\theta_{j}\right\}}_{j=1}^{s}$ and the number of sub-models *s*. In addition, it is also necessary to solve a classification problem: it is required to classify which part of the data is described by a sub-model rather than another one.

As reported in Nakada et al. (2005) and Lauer and Bloch (2019), in PWA system identification, a typical representation is the PWARX model. The general form of PWARX is given by

$$y_{k}=\left\{\begin{array}{c} \;\theta_{1}^{⊤}\left[\begin{array}{c} x_{k}\\ 1 \end{array}\right]+e_{k},\;if\;x_{k}\in X_{1}\;\\ \;\theta_{2}^{⊤}\left[\begin{array}{c} x_{k}\\ 1 \end{array}\right]+e_{k},\;if\;x_{k}\in X_{2}\;\\ \;\;\begin{array}{c} \vdots \end{array}\;\\ \;\theta_{s}^{⊤}\left[\begin{array}{c} x_{k}\\ 1 \end{array}\right]+e_{k},\;if\;x_{k}\in X_{s}\; \end{array}\right.$$ (7)

for (*k* = 1 … *N*), where $y_{k}\in R^{p}$ is the output vector and $e_{k}\in R^{p}$ is the noise at time *k*. Furthermore, the regression vector $x_{k}\in R^{n_{d}}$ can be defined by

$$x_{k}={\left[\begin{array}{c} y_{k-1}^{⊤}\;\ldots \;y_{k-n_{y}}^{⊤}\;u_{k-1}^{⊤}\;\ldots \;u_{k-n_{u}}^{⊤} \end{array}\right]}^{⊤}$$ (8)

where $u_{k}\in R^{m}$ is the input vector and *n*_*d*_ = *pn*_*y*_ + *mn*_*u*_, with non-negative integers *n*_*y*_ and *n*_*u*_ representing, respectively, the output and input lag orders (Nakada et al., 2005). Let $X\subseteq R^{n_{d}}$ be the regression space, and $X_{i}$ (*i* = 1, 2, *…* , *s*) represents a convex polyhedral subset of X . Each polyedron $X_{i}$ is assumed to satisfy $X_{i}=\left\{\;x_{k}\in X{:H}_{i}\;{\left[x_{k}\;1\right]}^{⊤}\leq 0\right\}$ , (*i* = 1, 2, *…* , *s*), $X_{i}\neq \emptyset \forall i\in \left\{1,2,\ldots ,s\right\},X_{i}\cap X_{j}=\emptyset \forall i,j\in \left\{1,2,\ldots ,s\right\},\;i\neq j$ , and ${⋃}_{i=1}^{s}X_{i}=X$ (Paoletti et al., 2007; Lauer and Bloch, 2019). Each row of $H_{i}$ defines a separating hyperplane between $X_{j}$ and the other regions (Lauer and Bloch, 2019). Indeed, if the PWA map is assumed to be continuous, the model parameters and the partition of the domain are not independent. At the switching surface between two modes, it must hold that (Lauer and Bloch, 2019)

$$\theta_{i}^{⊤}{\left[x_{k}\;1\right]}^{⊤}=\theta_{j}^{⊤}{\left[x_{k+1}\;1\right]}^{⊤},\;1\leq j<i\leq s$$ (9)

To be noted, assuming the presence of only two sub-dynamics for the PWARX model, the state points **x**_*k*_ belong either to $X_{1}$ or $X_{2}$ with just one separating hyperplane characterized by the normal vector $H_{1}$ . Hence, in this case, when the condition $H_{1}\;{\left[x_{k}\;1\right]}^{⊤}\leq 0$ holds, the dynamics is driven by the first subsystem. When the latter condition does not hold, switch to the second subsystem occurs, and the system evolves accordingly. In order to identify a PWARX model for the neuromuscular control, the first step requires to define the dimension of the regression space *n*_*d*_ and the lag orders *n*_*u*_ and *n*_*y*_ for the input and output data, respectively. It should be noted that while the output is *τ*_*c*_(*t*), the exogenous inputs that could be relevant to build the model are the sway angle *θ*(*t*) and the muscle RMS data. This great amount of exogenous information could lead to a non-parsimonious model identification. Thus, as suggested in Han et al. (2018), one can take advantage of the minimum redundancy maximum relevance (mRMR) principle to select the exogenous inputs, their lags, and the lags relative to the AR part of the model. The mRMR selection algorithm was formulated by Ding and Peng (2003) and further explained in Peng et al. (2005).

For each subject and trial, a large regression space is generated, delaying the input and output time series by a lag limit value equal to 20. Three large regression spaces, made by data relative to 7 subjects, are created for training, as follows: seven of ten regression spaces are concatenated for trials, namely, EO, EC, and DT, by preserving the lag coordinates. This corresponds to training–testing dataset split of 70%–30%. Each training set is shrunk with the mRMR algorithm to the five most relevant components for predicting the torques *τ*_*c*_ at the current time. Thus, by means of mRMR, the original large *n*_*d*_ training dataset is reduced to spaces with *n*_*d*_ = 5, where the lag selection of the AR and exogenous (X) components is driven directly by the data.

Each training dataset is partitioned through the spectral clustering approach, and the optimal number of clusters is evaluated through the silhouette method (Rousseeuw, 1987). Spectral clustering is employed to overcome the limitation of a parametric clustering approach, such as Gaussian mixture models, where the data are assumed to be a mixture of Gaussian distributions, by relying on a geometric approach (Ng et al., 2002). After clustering, data are labeled and employed to identify the polyhedral regions that partition the regression space. In order to do this, a soft margin support vector machine (SVM) is employed, as in Nakada et al. (2005).

After hyperplane identification, it remains to identify the ARX sub-models. Thus, possible data points lying on the hyperplanes are discharged, and the closed-form least mean square estimation based on the pseudo-inverse matrix is employed to recover the parameters of each sub-model (Ljung, 1999; Nakada et al., 2005). Normalized RMS error metric (NRMSE) is used to quantify the fitting goodness of the three identified models. Such measure will be referred to as fit percentage:

$$fit_{\%}=100\left(\;1-\frac{∥y-\hat{y}∥}{∥y-\mu_{y}∥}\;\right)$$ (10)

where *y* is the data output, $\hat{y}$ is the output produced by the model, and *μ*_*y*_ indicates the mean value of *y*. In order to test the reliability of the identification procedure in terms of robustness against overfitting, the above steps, maintaining the same identified lags, are applied using two additional dataset partitioning, i.e., 60%–40% and 50%–50%.

## 3 Results

In EC and DT conditions, the mRMR algorithm does not present the RMS of TA in the first five most relevant components (Table 1). To be noted, in the DT condition, mRMR selected three time lags of the control torque in the first five most significant components, thus suggesting a more AR scheme rather than those employed in the other two cases (i.e., characterized by two AR lags; Table 1).

**TABLE 1:** The five ARX components selected by the mRMR algorithm for the three augmented regression spaces namely, EO, EC, and DT, in terms of time-series lags chosen.

Condition	AR	X		
	*τ*_*c*_	*θ*	GA	TA
EO	{1; 20}	12	20	17
EC	{1; 20}	19	{17; 20}	
DT	{1; 4; 20}	18	19	

Note. ARX, autoregressive with exogenous input; mRMR, minimum redundancy maximum relevance.

**FIGURE 3:**
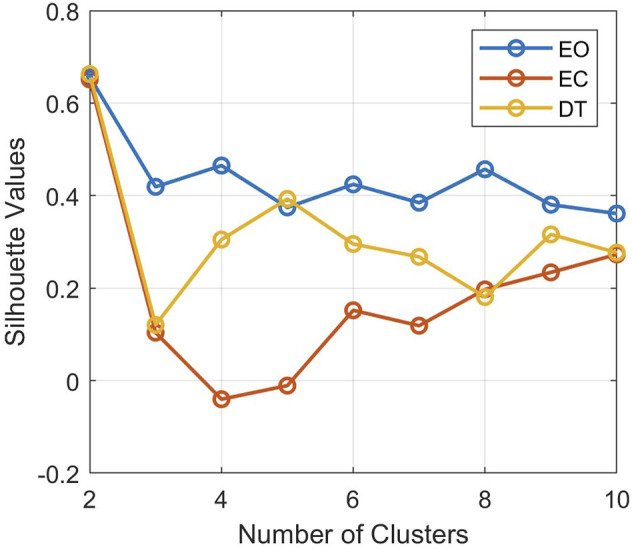
Silhouette spectral cluster evaluation for the three shrink regression spaces in eyes opened (EO), eyes closed (EC), and dual task (DT) conditions. Note that the optimal numbers of clusters is given in correspondence to the maximum of the silhouette values.

In all experimental conditions, the silhouette criterion suggested an optimal number of clusters equal to 2 (Figure 3). This is required to identify, in each condition, two ARX components and one boundary hyperplane dividing the two clusters. The three identified models are reported in Table 2.

**TABLE 2:** Regression vector (**x**_*k*_), estimated boundary hyperplane ( ${\hat{H}}_{12}$ ), and sub-models coefficients ( ${\hat{\theta }}_{1}$ and ${\hat{\theta }}_{2}$ ) for the identified models in the three conditions, i.e., EO, EC, and DT.

EO				EC				DT			
**x**_*k*_	${\hat{H}}_{12}$	${\hat{\theta }}_{1}$	${\hat{\theta }}_{2}$	**x**_*k*_	${\hat{H}}_{12}$	${\hat{\theta }}_{1}$	${\hat{\theta }}_{2}$	**x**_*k*_	${\hat{H}}_{12}$	${\hat{\theta }}_{1}$	${\hat{\theta }}_{2}$
*τ*_*ck*−1_	0.918	0.889	0.943	*τ*_*ck*−1_	3.470	0.912	0.929	*τ*_*ck*−1_	−2.250	1.275	1.196
*τ*_*ck*−20_	−4.574	0.010	-0.079	*τ*_*ck*−20_	−2.691	0.005	−0.105	*τ*_*ck*−20_	4.274	0.001	0.006
*TA*_*k*−17_	0.859	0.004	0.004	*GA*_*k*−17_	0.681	0.006	0.021	*GA*_*k*−19_	−0.805	−0.003	−0.001
*GA*_*k*−20_	2.078	0.003	−0.017	*GA*_*k*−20_	1.506	0.008	0.017	*θ*_*k*−18_	1.755	−0.022	−0.002
*θ*_*k*−12_	−2.472	0.005	−0.059	*θ*_*k*−19_	−3.460	0.008	0.029	*τ*_*ck*−4_	−0.960	−0.316	−0.238
	1.235	0.043	0.085		−0.340	0.030	0.082		−0.003	0.028	0.021

The identified models present adequate fitting properties in the training step, as highlighted by the value of the fit percentages for the three dataset partitioning (Table 3). However, it should be noted that subject 9 belonging to the testing data presented a moderate fit percentage value (about 70%) regarding the EC condition. All the other testing data guaranteed high NRMSE (Table 3).

**TABLE 3:** NMRSE fitting percentages obtained by the models identified for each experimental condition.

Condition	S1	S2	S3	S4	S5	S6	S7	S8	S9	S10
	Training data							Testing data		
EO	97.1	95.9	96.2	94.3	93.6	90.8	91.2	91.2	87.5	91.6
EC	95.8	94.8	93.9	95.9	95.5	89.5	93.1	93.1	67.9	92.0
DT	97.9	97.9	96.3	97.2	97.5	97.5	98.1	98.1	95.7	97.4
	Training data						Testing data			
EO	97.7	95.5	95.8	95.1	93.3	91.5	90.0	90.0	87.7	90.0
EC	96.0	94.5	93.3	96.4	96.0	89.9	92.1	92.1	69.4	91.7
DT	97.9	97.8	96.2	97.3	97.5	97.5	97.7	97.7	97.0	97.3
	Training data					Testing data				
EO	97.1	96.3	97.2	95.4	94.4	87.9	91.2	91.1	85.4	92.3
EC	95.7	95.1	94.5	95.9	96.7	87.3	93.0	92.9	72.4	91.9
DT	97.5	97.7	96.3	97.5	97.6	96.6	97.7	97.7	97.1	97.3

Note. The results are relative to three training–testing data split (70%–30%, 60%–40%, and 50–50%). S1, …, S10 indicate subjects.

## 4 Discussion

The findings of this study confirm that movement analysis techniques and dynamic posturography can be used to infer how the CNS handles sensory information and cognitive load while the subject undergoes sudden postural perturbations. Despite the control action being a postural reflex and should be attributed to lower control structures, part of the response may be attributed to higher-level CNS control structures (Horak and Nashner, 1986; Jacobs and Horak, 2007). This aspect is suggested by the criterion used to select the information and the time lags of the input–output data used to identify the models for the three different experimental conditions. Results indicate that the information content, useful to predict the control torque, markedly changed for the three tasks (Table 1). Indeed, all the exogenous information in the EO condition can be used to obtain good fits in both the training and testing phases (Table 3). Moreover, the lag orders of the exogenous signals, i.e., *θ*, GA, and TA, are always greater or equal to 12. This means that the delays used to obtain a parsimonious model of the physiological control torque are of the order of 120–200 ms, comparable with physiological values reported for upright stance models (Peterka, 2002; Bottaro et al., 2005). It should also be noted that both muscles are relevant in the EO condition, while for EC and DT, the TA time-lagged muscle activity was not ranked within the first 5 regressors considered for the identification procedure. More precisely, in the EC and DT conditions, two GA time lags were identified to be meaningful in modeling the control action (Table 1), while TA did not appear, eventually supporting that sensory deprivation or cognitive loads can steer the CNS to actuate different control schemes.

**FIGURE 4:**
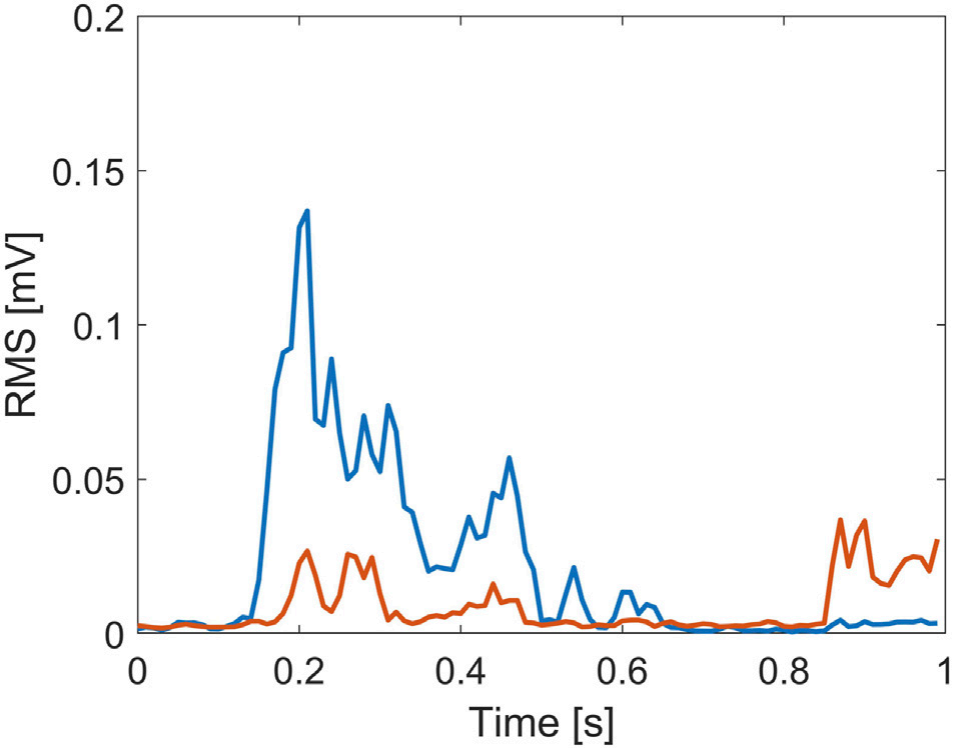
Root mean square (RMS) value of the myoelectric activity for the tibialis anterior (red line) and gastrocnemius (blue line) recorded during the EO trial, from a representative subject.

**FIGURE 5:**
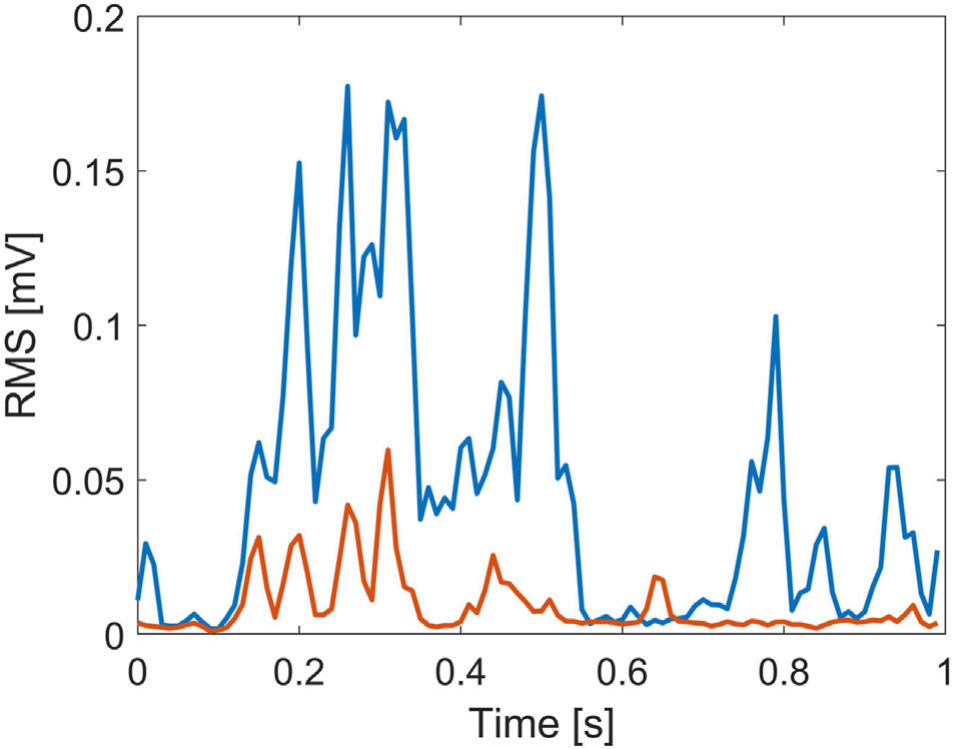
Root mean square (RMS) value of the myoelectric activity for the tibialis anterior (red line) and gastrocnemius (blue line) recorded during the EC trial, from a representative subject.

Due to the nature of the experiment, the gastrocnemius is expected to play a key role, since the support base moved backward with respect to the visual field. Hence, GA needs to activate in order to prevent the subject from falling forward (Horak et al., 1989). This is supported by considering that the GA information is taken into account in all three models (see Section 3). However, its role seems to be further enhanced in EC condition: the selection of two time lags for the GA component and the exclusion of TA in EC rather than EO might indicate that the CNS employed the visual sensory information to regulate more efficiently the redundancy of the control structure (Mengarelli et al., 2019). This explanation is consistent with Albertsen et al. (2017), where it is highlighted that postural adjustment in EC might be promoted by joint stiffening or increment of postural proprioceptive or vestibular reflex responses, while in the presence of vision, the CNS could afford low degrees of muscle stiffness, which might no longer be possible in visual deprivation (Albertsen et al., 2017). To be noted, the lack of TA in the identified models in EC and DT conditions does not mean that the role of tibialis is negligible for balance recovery in the EC condition since a modulation of the response involving both the ankle muscles is well-acknowledged (Perucca et al., 2014). Indeed, both muscles are involved in the balance maintenance process in both conditions (Figures 4, 5). However, the predominant role of the GA with respect to TA in EC condition seems to be supported by considering the myoelectric activity of both muscles within the time epoch considered in this study, where the ratio between GA and TA area under the curve (Campbell et al., 2009) resulted in 2.48 ± 0.43 (EO) and 5.02 ± 0.55 (EC), averaged over all the participants.

Albeit EO and EC conditions promoted models that differed mainly in managing exogenous information, they presented a similar ARX structure, i.e., with the same number of AR components (see Section 3). This cannot be stated for the DT model (Table 1), for which an increase in AR order is captured if compared with the other two experimental conditions. Such structural change could be partially explained by the involvement of higher CNS control centers in a cognitive task, leading to a more reflex-based control. Indeed, the additional AR term presented a lag order of 4, (40 ms), which indicates a sudden disturbance compensation. From a physiological point of view, this could be explained by greater involvement of the proprioceptive information to produce a postural adjustment (low-level scheme), thus suggesting a control policy mainly driven by the peripheral information. This is not completely surprising since it has been reported that introducing torque-related information for balance regulation could be a reasonable physiological regulatory strategy (Peterka, 2009, 2018).

**FIGURE 6:**
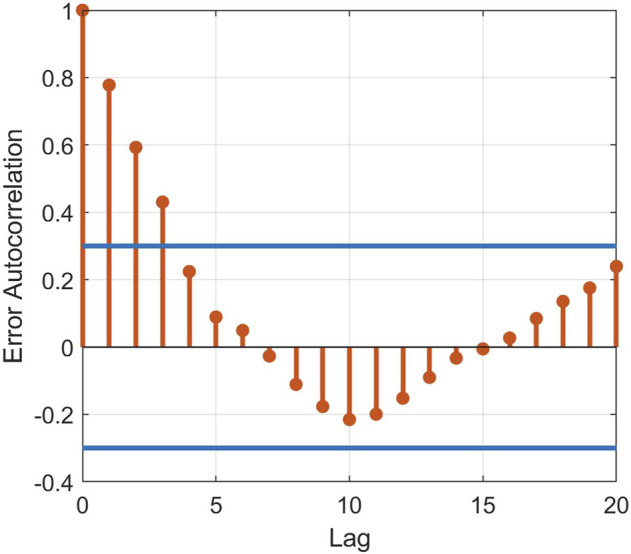
Error signal autocorrelation for a representative subject. The error signal was obtained as the difference between the output generated by the model and the measured torque.

Another aspect that should be underlined is the number of sub-dynamics that silhouettes identified (Figure 3). The number of clusters remained equal to two for all the three different experimental trials and the lagged signals, suggesting an akin control response between the considered perturbed conditions. Although a clear interpretation of the subdivision of the five-dimensional data in two clusters is not straightforward, due to the black-box nature of the identification procedure, the models guaranteed satisfactory autocorrelation of the error signals (Figure 6) and fitting percentage in both training and testing (Table 3). This supports the generalization properties of the identification procedure, also by considering that no detriment is observed by progressively increasing the amount of testing data (up to 50%) at the expense of lower data available for training (Table 3). Note that in the present study, with time lag equal to 20 and given four regressors, i.e., sway angle, torque, and myoelectric activity of two shank muscles (tibialis and gastrocnemius), the total number of possible regressors was equal to 80. Therefore, we choose *n*_*d*_ = 5 as a fixed value for two reasons: first, it guaranteed a robust fitting of the testing data even when the size of the training set was reduced, thus avoiding overfitting issues (Table 3). On the other hand, *n*_*d*_ = 5 allowed to obtain better interpretable models with respect to system identification approaches, e.g., neural networks or deep learning, for which the goodness of fitting is favored with respect to the model interpretability (Ljung et al., 2020).

**FIGURE 7:**
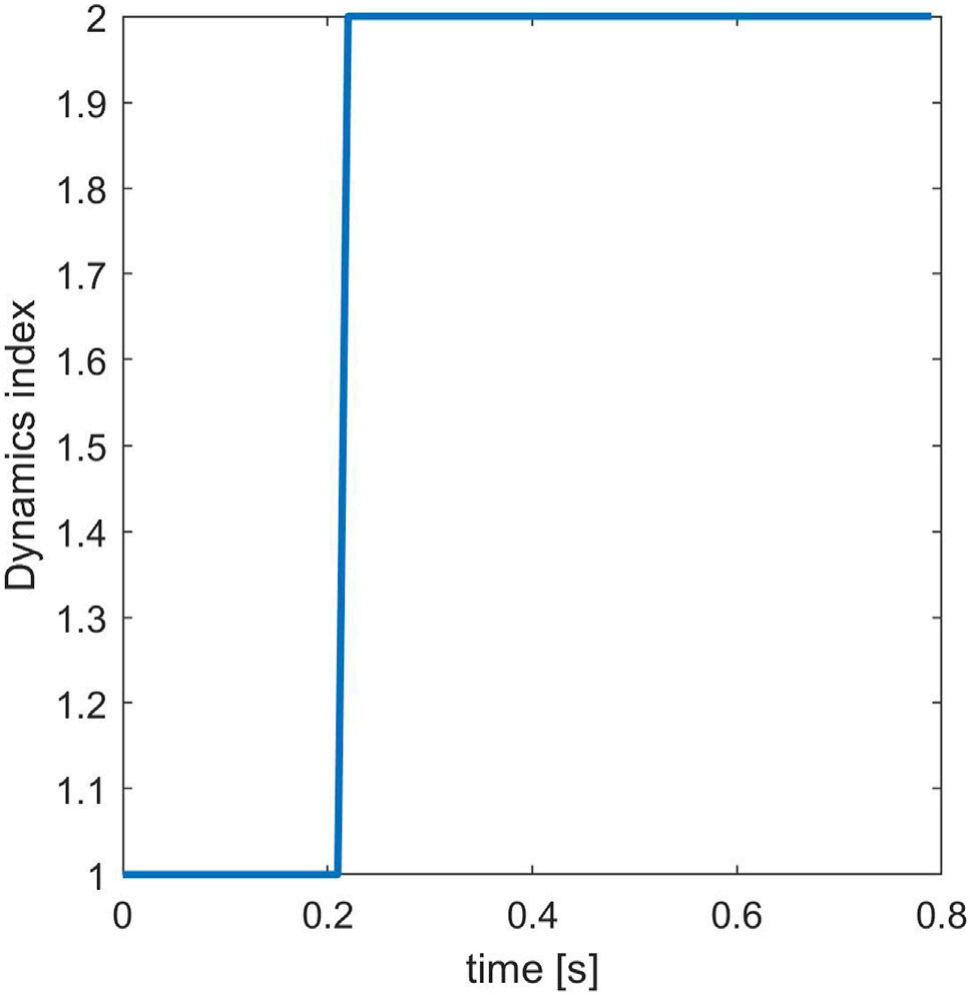
Switching signal of a representative subject in DT condition. Dynamic index i corresponded to the *i*th autoregressive with exogenous input (ARX) dynamic fitted with the *i*th data cluster, for *i* = 1,2. The signal shows one switch, and in this case, the second dynamic occurred at 230 ms.

Moreover, by the analysis of the switching signals between the two sub-dynamics, obtained considering the training data, it can be observed that the models present one switch (Figure 7), which occurs at 210, 260, and 230 ms for OA, OC, and DT, respectively (median values among all the subjects). The aforementioned values are always lower than about 300 ms, which is approximatively the time when the platform ended to move. Thus, the identified neuromuscular control models work with a certain control policy in the first part of the experiment, where the inertial forces vary more due to the presence of the support base acceleration (3) and switches to another policy when the inertia is reduced (after 300 ms), eventually confirming that the data partitions are consistent with the process dynamics. This suggests that the models made by two ARX components capture a significant amount of information, confirming the line proposed in Corradini et al. (1997) regarding the capability of black-box modeling to be sensitive to balancing strategy changes. Hence, such identification procedures can be employed concurrently with classical posturographic analyses to investigate the neural policies adopted for maintaining an upright stance challenged by external disturbances.

In the present study, both biomechanical models and controllers are not linearly approximated, despite that in many works related to the characterization of the postural control this represents a common procedure (Peterka, 2000; Corradini et al., 1997; Engelhart et al., 2014). However, as reported in Section 2, this is mainly due to the nature of the experiment, since the perturbation magnitude is higher than the physiological postural noise (Conforto et al., 2001), thus making the linearization hypothesis an over-simplification of the problem. In passing, the perturbation was appropriate to elicit a balance response relying mainly on the ankle joint, since the magnitude of the disruption was within the range reported by Diener et al. (1988), who observed a counterbalancing response that relied almost entirely on the variation of the ankle strategy amplitude alone. Further confirmation is found in the ARX sub-model coefficients: for a given predictor, a change of the magnitude of the sign or of both is obtained (see Section 3), indicating that unimodal linear controllers may be not adequate to fit postural data obtained through support base movement perturbation. Nevertheless, a piecewise approach seems to be adequate to successfully model the process when the eventual aim is the analysis from a physiological viewpoint. Indeed, despite other kinds of models, such as nonlinear ARX (NARX) or PWNARX, could indeed be applied, the potential benefits in terms of data fitting would be obtained at the cost of poorer and more difficult interpretability of the results, hindering their practical use in the clinical context. Moreover, it is noteworthy that despite the single-link inverted pendulum representing a simplified model of the human upright stance, it appears suitable for modeling stance maintenance in perturbed conditions. Indeed, many previous studies investigated balance responses to external perturbations relying on single-link inverted pendulum model, where the ankle joint is the only actuated joint for counteracting the disruption (Van Der Kooij and De Vlugt, 2007; Peterka, 2009; Davidson et al., 2010; Schut et al., 2020). In addition, this kind of model for the human upright stance constitutes the core of any balancing task, also in dynamical conditions, where a multi-link structure is often reduced to the CoP–COM relation (Chevallereau et al., 2008; Morasso, 2020).

It deserves to be pointed out that discontinuous systems (Orlov, 2008) with multiple dynamics were employed to model upright maintenance in perturbed (Tigrini et al., 2019) and unperturbed conditions, with internal physiological noise sources (Bottaro et al., 2005; Suzuki et al., 2012; Nomura et al., 2013). The latter resulted in highly interpretable and constitute a great advance toward the understanding of neuromuscular control CNS actuates in posture maintenance. In the PWARX identification context, the idea of multiple dynamics with possible discontinuities is still preserved; thus, the high fits obtained in this study seem to confirm the hypothesis that neuromuscular control of the upright stance can be modeled as a multi-dynamic process (Suzuki et al., 2012; Nomura et al., 2013). The use of PWARX in modeling neuromuscular control merits further investigations, and additional efforts will be devoted to the investigation of the entire body chain, whose employment would provide valuable additional insights regarding the dynamics of balance response to a sudden external perturbation. These aspects should be taken into account in future studies, since they require the use of different system identification approaches, e.g., multi-input multi-output, and the acquisition of kinematics and myoelectric activity also from the upper segments of the human body. Eventually, such kind of system identification framework can be also applied in posturographic data analysis and other motor tasks, e.g., gait, in relation to different experimental conditions or populations, such as elderly and pathological individuals. A further possible research line encompasses the investigation of additive sub-dynamics, possibly involving voluntary control efforts, which can manifest themselves over longer temporal epochs.

## 5 Conclusion

In the present work, the PWARX framework was employed to identify different models of neuromuscular control of upright stance maintenance with respect to three different experimental perturbations, commonly used in clinical and research scenarios. The PWARX family and the identification procedure led to parsimonious models that captured the differences present in the three experimental settings, confirming that black-box modeling can be used concurrently with dynamic posturography analysis, supporting physicians in clinical evaluations and interpretation.

## Data Availability

The raw data supporting the conclusions of this article will be made available by the authors, without undue reservation.
